# TGR5-HNF4α axis contributes to bile acid-induced gastric intestinal metaplasia markers expression

**DOI:** 10.1038/s41420-020-0290-3

**Published:** 2020-07-06

**Authors:** Zhen Ni, Yali Min, Chuan Han, Ting Yuan, Wenquan Lu, Hassan Ashktorab, Duane T. Smoot, Qiong Wu, Jian Wu, Weizheng Zeng, Yongquan Shi

**Affiliations:** 1 State Key Laboratory of Cancer Biology and Institute of Digestive Diseases, Xijing Hospital, Fourth Military Medical University, Xi’an, Shaanxi 710032 China; 2 Department of Gastroenterology, General Hospital of Western Theater Command, Chengdu, Sichuan 610083 China; 3 Department of Gastroenterology, Second Affiliated Hospital of Xi’an Medical College, Xi’an, Shaanxi 710038 China; 4 Department of Endocrinology, General Hospital of Western Theater Command, Chengdu, Sichuan 610083 China; 5 Department of Gastroenterology, 989 Hospital of the People’s Liberation Army, Luoyang, Henan 471003 China; 6 Department of Gastroenterology, First Affiliated Hospital of Zhengzhou University, Zhengzhou, Henan 450052 China; 7 Department of Medicine and Cancer Center, Howard University, Washington, DC 20060 USA; 8 Department of Internal Medicine, Meharry Medical College, Nashville, TN 37208 USA

**Keywords:** Cell signalling, Stomach diseases

## Abstract

Intestinal metaplasia (IM) increases the risk of gastric cancer. Our previous results indicated that bile acids (BAs) reflux promotes gastric IM development through kruppel-like factor 4 (KLF4) and caudal-type homeobox 2 (CDX2) activation. However, the underlying mechanisms remain largely elusive. Herein, we verified that secondary BAs responsive G-protein-coupled bile acid receptor 1 (GPBAR1, also known as TGR5) was increased significantly in IM specimens. Moreover, TGR5 contributed to deoxycholic acid (DCA)-induced metaplastic phenotype through positively regulating KLF4 and CDX2 at transcriptional level. Then we employed PCR array and identified hepatocyte nuclear factor 4α (HNF4α) as a candidate mediator. Mechanically, DCA treatment could induce HNF4α expression through TGR5 and following ERK1/2 pathway activation. Furthermore, HNF4α mediated the effects of DCA treatment through directly regulating KLF4 and CDX2. Finally, high TGR5 levels were correlated with high HNF4α, KLF4, and CDX2 levels in IM tissues. These findings highlight the TGR5-ERK1/2-HNF4α axis during IM development in patients with BAs reflux, which may help to understand the mechanism underlying IM development and provide prospective strategies for IM treatment.

## Introduction

Gastric cancer (GC) remains one of the most prevalent malignancies worldwide^1^. As a precancerous lesion, intestinal metaplasia (IM) significantly increases the risk of GC^2,3^. Although phenotypic changes and histopathological characteristics are relatively well-understood, the underlying pathogenesis of IM remains obscure.

*Helicobacter pylori* (Hp) infection is an established etiologic factor in gastric carcinogenesis^4^. However, Hp eradication cannot reverse IM phenotype and reduce the risk of GC in patients with IM^5^. Thus, pathogenic factors other than Hp infection may play important roles in such settings. Previous studies suggested that duodenogastric reflux (DGR) contributes to IM and subsequent GC development^6^. Clinical researches indicated that bile acids (BAs) concentrations in gastric juice were positively correlated with the degree of IM regardless of Hp infection^7^, both in antrum^8^ and cardia^9^. Our previous study first uncovered that BAs exposure could significantly induce gastric epithelial cells columnar genes expression through microRNA–mRNA networks involving a miR-92a-5p/FOXD1/nuclear factor-κB (NF-κB) axis^10^. These results verified the key role of BAs reflux in gastric IM initiation and progression. However, the underlying mechanisms remain largely unknown.

Intestine developmental signaling pathways reactivation is involved in metaplastic phenotype after pathogenic factors exposure. Kruppel-like factor 4 (KLF4) and caudal-type homeobox 2 (CDX2) are the fundamental transcription factors (TFs) in enterocyte differentiation and maturation^11,12^. Stomach characters loss and intestine features acquisition have been demonstrated in both IM tissues and transgenic mice^13–15^. We previously demonstrated that BAs exposure could significantly increase KLF4 and CDX2, and simultaneously inhibit SRY-box 2 expression^10,16,17^. These results indicate that aberrant developmental programs are involved in pathogenic effects of BAs exposure. However, the key events mediating BAs effects and orchestrating KLF4 and CDX2 upregulation in gastric IM development have not been fully clarified.

In the current study, we focused on G-protein-coupled BA receptor 1 (GPBAR1, also known as TGR5), a key receptor that mediated both physiological and pathological effects of secondary BAs. We demonstrated that TGR5 was involved in BA-induced metaplasia process via hepatocyte nuclear factor 4α (HNF4α) activation. Further, we elucidated that HNF4α contributed significantly to BA-induced columnar genes expression through directly regulating KLF4 and CDX2. Our findings revealed an important role of TGR5-HNF4α axis in intestine reprogramming caused by chronic BAs reflux in gastric epithelium and subsequent progression of IM.

## Materials and methods

### Reagents and chemicals

Deoxycholic acid (DCA), SB756050, U0126, SB239063, and MK2206 were purchased from MedChemExpress (Shanghai, China). BI6015 was purchased from Cayman Chemical (Ann Arbor, Michigan, USA). Dimethyl sulfoxide (DMSO) was purchased from Sigma-Aldrich (St. Louis, MO, USA).

### Cell culture and treatment

The human normal gastric epithelial cell line (GES-1) and gastric carcinoma cell lines (AGS, MKN45, BGC823, AZ521, N87, KATO III, and SGC7901) were originally purchased from American Type Culture Collection (ATCC) and maintained in our laboratory. The normal human gastric epithelial cell line HFE-145 was developed and kindly provided by Professor Hassan Ashktorab and Professor Duane T. Smoot. All cell lines were cultured at 37 °C in a humidified atmosphere of 5% CO_2_ in RPMI 1640 medium (Thermo Scientific, Waltham, MA, USA) supplemented with 10% fetal bovine serum (Biological Industries, Kibbutz Beit Haemek, Israel) and 1% penicillin–streptomycin solution (Thermo Scientific, Waltham, MA, USA). All cell lines were authenticated by Short Tandem Repeat (STR) DNA profiling and were tested negative for mycoplasma contamination. For BAs treatment, the cells were seeded into 6 cm culture dishes. After reaching ~60–70% confluence, the cells were starved for 24 h and then treated with DCA dissolved in DMSO at the indicated concentrations for different times in medium without fetal bovine serum. For pathway blocking, the cells were pretreated with inhibitors dissolved in DMSO for 1 h before DCA treatment. The negative control was treated with DMSO.

### Total RNA extraction and quantitative real-time RT-PCR

Total RNA was extracted using the RNeasy Mini Kit (QIAGEN, Hilden, Germany) according to the manufacturer’s instructions. In total, 500 ng RNA was synthesized into cDNA using the PrimeScript RT reagent kit (TaKaRa, Shiga, Japan) and Mir-X mRNA First-Strand Synthesis Kit (TaKaRa) in a 10 μL volume. Real-time PCR was performed on a CFX96 system using TB Green Premix Ex Taq II (TaKaRa) with 2 μL cDNA and 0.8 µL primers in a final volume of 20 μL. The final PCR conditions were as follows: pre-denaturation at 95 °C for 10 min, followed by 44 cycles at 95 °C denaturation for 10 s, 60 °C annealing for 20 s, and 72 °C extension for 10 s. The target gene mRNA was normalized to human glyceraldehyde 3-phosphate dehydrogenase (GAPDH) and calculated using the 2^−ΔΔCT^ method. Primer sequences are shown in Table 1.

**Table 1:** The sequences of primers used in this study.

Gene symbols	Primers	5′−3′
*HNF4α*	Forward	GTTCAAGGACGTGCTGCTCCTA
	Reverse	AGGCATACTCATTGTCATCGATCTG
*CDX2*	Forward	TTCACTACAGTCGCTACATCACCA
	Reverse	CTGCGGTTCTGAAACCAGATT
*KLF4*	Forward	AAGAGTTCCCATCTCAAGGCACA
	Reverse	GGGCGAATTTCCATCCACAG
*ALPI*	Forward	CATTCCAGGTCACCAGATCCA
	Reverse	AGAAATCTATGCCCAGCATCCAG
*MUC13*	Forward	AGAAACATTCCATGGCCTATCAAGA
	Reverse	CTTGTCATCAGCACGCATTTCA
*Villin1*	Forward	CGACTGCTACCTGCTGCTCTACAC
	Reverse	CGGCTTGATAAGCTGATGCTGTAA
*MUC5AC*	Forward	CTGACCAAGGGCTCCGTC
	Reverse	GCAAGGGTGTGGCAGACG
*GAPDH*	Forward	GCACCGTCAAGGCTGAGAAC
	Reverse	TGGTGAAGACGCCAGTGGA
*KLF4* Promoter	Forward	GGCGGAGTCCCGCGCCGAAC
	Reverse	TCAAAAAGTGCACACCGAGCC

*ALPI* alkaline phosphatase, intestinal, *CDX2* caudal-type homeobox 2, *HNF4α* hepatocyte nuclear factor 4α, *KLF4* Kruppel-like factor 4, *MUC13* mucin 13, *MUC5AC* mucin 5AC.

### Western blotting

Cells were collected and total protein was extracted using RIPA cell lysis buffer (Beyotime Biotechnology, Shanghai, China) with protease and phosphatase inhibitor cocktails (MedChemExpress, Shanghai, China). Protein was quantified using the bicinchoninic acid method according to the manufacturer’s instructions (Thermo Scientific, Waltham, MA, USA). Proteins (20–30 μg) were electrophoresed on a 8–12% polyacrylamide gel, transferred onto a nitrocellulose membrane (Pall Corporation, Port Washington, NY, USA) at 25 V for 35 min, and blocked for 1 h in 10% non-fat milk in 1× Tris Buffered Saline (TBS)/0.1% (v/v) Tween-20 at room temperature. Primary antibodies were added and incubated overnight at 4 °C. Human HNF4α (#3113, 1:1000), CDX2 (#12306, 1:1000), Villin1 (#2369, 1:1000), p21 (#2947, 1:1000), and cadherin-17 (CDH17) (#42919, 1:1000) were from Cell Signaling Technology (Danvers, MA, USA). Primary antibodies against human KLF4 (ab215036, 1:1000), TGR5 (ab72608, 1:1000), mucin 5AC (MUC5AC) (ab24071, 1:1000), and mucin 13 (MUC13) (ab235450, 1:1000) were from Abcam (Cambridge, UK). Primary antibody against human intestinal alkaline phosphatase (ALPI) (A6226, 1:1000), and β-actin (AC006, 1:4000) were from ABclonal (Wuhan, China). Second antibodies (horseradish peroxidase-conjugated anti-mouse/rabbit IgG, 1:4000, Cell Signalling Technology) were incubated at room temperature for 1 h. Signals were detected using WesternLumaxLight Sirius HRP substrate reagent (ZETA-Life, San Francisco, CA, USA). All data were normalized to human β-actin. The bands were scanned using a ChemiDocXRS+ Imaging System (Bio-Rad, Hercules, CA, USA) and quantified using Image Lab v5.2 software (Bio-Rad).

### Tissues collection and immunohistochemistry

This study was approved by the Institutional Ethics Committee of First Affiliated Hospital of Fourth Military Medical University. Formalin-fixed, paraffin-embedded normal and IM biopsy specimens from stomach antrum were obtained from the pathology department of Xijing Digestive Disease Hospital. All patients were *H. pylori* negative confirmed by rapid urease test or ^13^C-breath test. All samples were diagnosed by hematoxylin and eosin staining by at least two pathology experts and were classified into normal stomach tissues, mild IM (goblet cells percentage <1/3), moderate IM (goblet cells percentage 1/3–2/3), and severe IM (goblet cells percentage ≥2/3) according to the proportion of the gastric glands being replaced by the metaplastic issue^18,19^. Paraffin-embedded consecutive slides of gastric disease tissue microarrays (ST8017a, ST806, and IC00011b) including 67 cases of chronic superficial gastritis and 120 cases of IM were purchased from Alenabio (Xi’an, China).

Immunohistochemistry (IHC) staining was performed for TGR5, HNF4α, P1-HNF4α, P2-HNF4α, CDX2, and KLF4 in stomach tissues using the standard Biotin-Streptavidin HRP Detection Kit (Zsbio, Beijing, China). Briefly, tissue slides were dewaxing and hydration using dimethylbenzene and ethanol. Next, 3% H_2_O_2_ was used to eliminate endogenous peroxidase activity for 10 min at room temperature. Antigen retrieval was carried out by heat treatment in 1× citrate buffer for 2 min and then cooling the sections to room temperature. Primary antibodies against human HNF4α (#3113, 1:400, Cell Signaling Technology), KLF4 (ab215036, 1:1000, Abcam), CDX2 (#12306, 1:100, CST), P1-HNF4α (ab41898, 1:100, Abcam), P2-HNF4α (PP-H6939-00, 1:100, R&D), and TGR5 (ab72608, 1:300, Abcam) were incubated overnight at 4 °C. A secondary antibody (1:400) was added and incubated for 30 min at room temperature. Diaminobenzidine (DAB) reagent was added for 1–3 min at room temperature. Finally, the slides were rinsed with running water and counterstained with hematoxylin for 2 min. A concentration-matched nonspecific rabbit IgG was used as a control.

The slides were scanned and viewed using Pannoramic Viewer (3DHISTECH, Ltd, Budapest, Hungary). The staining intensity of HNF4α, CDX2, and KLF4 was semi-quantitatively determined using the H-score method^20^. Only nuclei stained unequivocally were considered as positive. An *H*-score ≥ 50 is considered positive. TGR5 expression was semi-quantitatively determined according to the staining intensity and percentage of positive cells^21^. IHC scores < 6 and ≥6 were considered as low and high expression, respectively.

### Immunofluorescence

To detect the expression of HNF4α, CDX2, and KLF4 induced by DCA treatment, 3 × 10^4^ GES-1 cells were cultured on a Millicell EZ SLIDE 4-well glass slide (Merck, Darmstadt, Germany). On the next day, the cells were starved for 24 h and then treated with DCA for another 24 h. The cells were fixed with 4% paraformaldehyde for 30 min and washed with phosphate-buffered saline (PBS) three times. The cells were treated with 0.5% Triton-X 100 for 30 min and then blocked with blocking solution (Hat Biotechnology, Xi’an, China) for 30 min at room temperature. The cells were stained with primary antibodies against HNF4α (#3113, 1:400, CST), KLF4 (ab215036, 1:100, Abcam), and CDX2 (#12306, 1:50, CST) overnight at 4 °C. After washing with 1× PBS/0.1% (v/v) Tween-20 three times, the cells were incubated with Alexa Fluor 594-conjugated secondary antibodies (1:250, Yeasen Biotech, Shanghai, China) at room temperature for 1 h. Cell nuclei were stained with 4′,6-diamidino-2-phenylindole (1:500, CST) for 5 min. Fluorescence was scanned using a Nikon A1R Confocal Microscope (Tokyo, Japan).

### RT2 profiler PCR array analysis of GES-1 cells treated with and without DCA

Total RNA of GES-1 cells treated with and without 200 μM DCA for 24 h in a 6-well culture plate was extracted using an RNeasy Mini Kit (Qiagen) according to the manufacturer’s instructions. RNA was purified and cDNA was synthesized from 1 μg total RNA using the RT2 First Strand Kit (Qiagen) according to the manufacturer’s instructions. Human TFs RT2 Profiler PCR Arrays (#PAHS-075ZA, Qiagen) were used for TFs profiling using the CFX96 system. PCR array data were analyzed according to the manufacturer’s instructions using a Microsoft Excel Template available from the manufacturer’s website. Each array contained five housekeeping genes (*ACTB*, *B2M*, *GAPDH*, *HPRT1*, and *RPLP0*) against which the sample data were normalized. The transcript level of each gene was quantified according to the 2^−ΔΔCT^ method. CT values > 35 were not included in the analysis and considered as negative.

### Cell transfection

The human HNF4α2 (NM_000457) and HNF4α8 (NM_175914) overexpression lentiviral was constructed by GeneCopoeia (Rockville, MD, USA). TGR5 (NM_001077191) and CDX2 (NM_001265) overexpression lentiviral and short hairpin RNAs (shRNAs) against human HNF4α and CDX2 (Table 2) were designed and constructed by GeneChem (Shanghai, China). Small interfering RNA (siRNA) duplexes against human TGR5 and KLF4 were designed and constructed by GenePharma (Shanghai, China) (Table 2).

**Table 2:** The sequences of shRNAs and siRNAs used in this study.

Gene symbols	Sequences	5′−3′
Human *HNF4α*	Sense	CGAGCAGATCCAGTTCATCAA
	Antisense	TTGATGAACTGGATCTGCTCG
Human *KLF4-1*	Sense	CCUUACACAUGAAGAGGCATT
	Antisense	UGCCUCUUCAUGUGUAAGGTT
Human *KLF4-2*	Sense	CAGCCAGAAAGCACUACAATT
	Antisense	UUGUAGUGCUUUCUGGCUGTT
Human *CDX2*	Sense	AGCCCTTGAGTCCGGTGTCTT
	Antisense	AAGACACCGGACTCAAGGGCT
Human *TGR5-1*	Sense	CCUGUACCUCGAAGUCUAUTT
	Antisense	AUAGACUUCGAGGUACAGGTT
Human *TGR5-2*	Sense	GUCGACCUGGACUUGAACUTT
	Antisense	AGUUCAAGUCCAGGUCGACTT
Human *TGR5-3*	Sense	UCGUCUACUUGGCUCCCAATT
	Antisense	UUGGGAGCCAAGUAGACGATT
Negative control	Sense	UUCUCCGAACGUGUCACGUTT
	Antisense	ACGUGACACGUUCGGAGAATT

*CDX2* caudal-type homeobox 2, *HNF4α* hepatocyte nuclear factor 4α, *KLF4* Kruppel-like factor 4, *shRNAs* short hairpin RNA, *siRNAs* small interfering RNAs, *TGR5* G-protein-coupled bile acid receptor 1.

To establish stable transfection cell lines, GES-1 and AGS (1 × 10^5^) cells were infected with concentrated lentiviral stock at ~50 multiplicity of infection at a final concentration of 0.5 μg/mL polybrene for 10 h at 37 °C in 24-well culture plates. After transferring the cells into 25 cm^2^ flasks, a final concentration of 1 µg/mL puromycin was added to remove uninfected cells. The cell culture medium was replaced every 2–3 days with fresh and puromycin-containing RPMI 1640.

For transient transfection, after reaching ~70% confluence in a six-well culture plate, the cells were transfected with 150 nmol of siRNA plasmid with 7.5 μL of EndoFectin™ Max (GeneCopoeia) transfection reagent in 1.5 mL Gibco™ Opti-MEM™ medium for 10 h at 37 °C. The medium was replaced with fresh RPMI 1640 medium. A scrambled sequence was used as a negative control. Target genes were examined at 72 h after transfection using western blotting (WB).

The effect of DCA on HNF4 transcription activity was examined using HNF4 transcriptional response element (HNF4 TRE) plasmids with a Gaussia Luciferase reporter designed and constructed by GeneCopoeia. Briefly, GES-1 cells were seeded into a six-well culture plate. After reaching ~70% confluence, the cells were transfected with 2 μg HNF4 TRE or negative control plasmids with 10 μL of EndoFectin™ Max transfection reagent in 2 mL Gibco™ Opti-MEM™ medium for 10 h at 37 °C. Twenty-four hours after transfection, the cells were treated with 200 μM DCA for another 24 h. Next, 100 μL supernatant was collected and Gaussia Luciferase activity was detected using a Secrete-Pair Gaussia Luciferase Assay Kit (GeneCopoeia) according to the manufacturer’s instructions.

### Dual-luciferase reporter assays

Briefly, 2000 bp fragments of the human KLF4 and CDX2 promoter were obtained from Ensembl and predicted in the JASPAR database (http://jaspar.binf.ku.dk). The wild-type and corresponding mutational KLF4 promoter fragments covering the HNF4-binding site and CDX2 promoter fragments covering the HNF4-binding sites were PCR-amplified and cloned into the firefly luciferase reporter plasmid pGL3-basic vector (Promega, Madison, WI, USA). Luciferase activity was then detected using a Dual-Luciferase Reporter assay kit (Promega) at 48 h after reporter transfection by Lipofectamine 2000 transfection reagent (Invitrogen, Carlsbad, CA, USA) in GES-1 or AGS cells. Firefly luciferase activity was normalized to *Renilla* luciferase activity and the final data were presented as the fold induction of luciferase activity compared with that of the negative control.

### Chromatin immunoprecipitation

Chromatin immunoprecipitation (ChIP) assays were performed using the EZ ChIP™ ChIP Kit (Millipore, Billerica, MA, USA). The cells were cross-linked with 1% formaldehyde for 10 min at 37 °C and quenched with 2.5 M glycine for 5 min at room temperature. DNA was immunoprecipitated from the sonicated cell lysates using HNF4α antibody (Abcam) and subjected to PCR to amplify the HNF4-binding site (Table 1). The amplified fragments were analyzed on an agarose gel. A nonspecific antibody against IgG served as a negative control.

### Statistical analysis

All cell culture experiments were performed in triplicate to reduce error and ensure reproducibility. All quantitative data were expressed as the means ± SEM. Differences between two groups were examined using two-tailed Student’s *t*-test. Differences between multiple groups were compared by one-way analysis of variance with Dunnett’s post hoc tests. All categorical data were expressed as rates. Differences between two groups were examined by *χ*^2^-test. The correlation between HNF4α, CDX2, and KLF4 expression was examined using Spearman’s correlation analysis. Statistical analysis was performed using SPSS 13.0 software (SPSS, Inc., Chicago, IL, USA) or GraphPad Prism 5.0 (GraphPad Software, San Diego, CA, USA). *P* < 0.05 was considered statistically significant (*P < 0.05, **P < 0.01).

## Results

### DCA treatment induced metaplasia markers expression in gastric epithelial cells

Our previous results have demonstrated that primary BAs chenodeoxycholic acid (CDCA) treatment could significantly induce metaplasia markers expression in vitro^10,16^. Clinical researches indicate that second BAs, especially DCA, are more toxic and are the predominant BAs refluxate in stomach^22^.

**Fig. 1:**
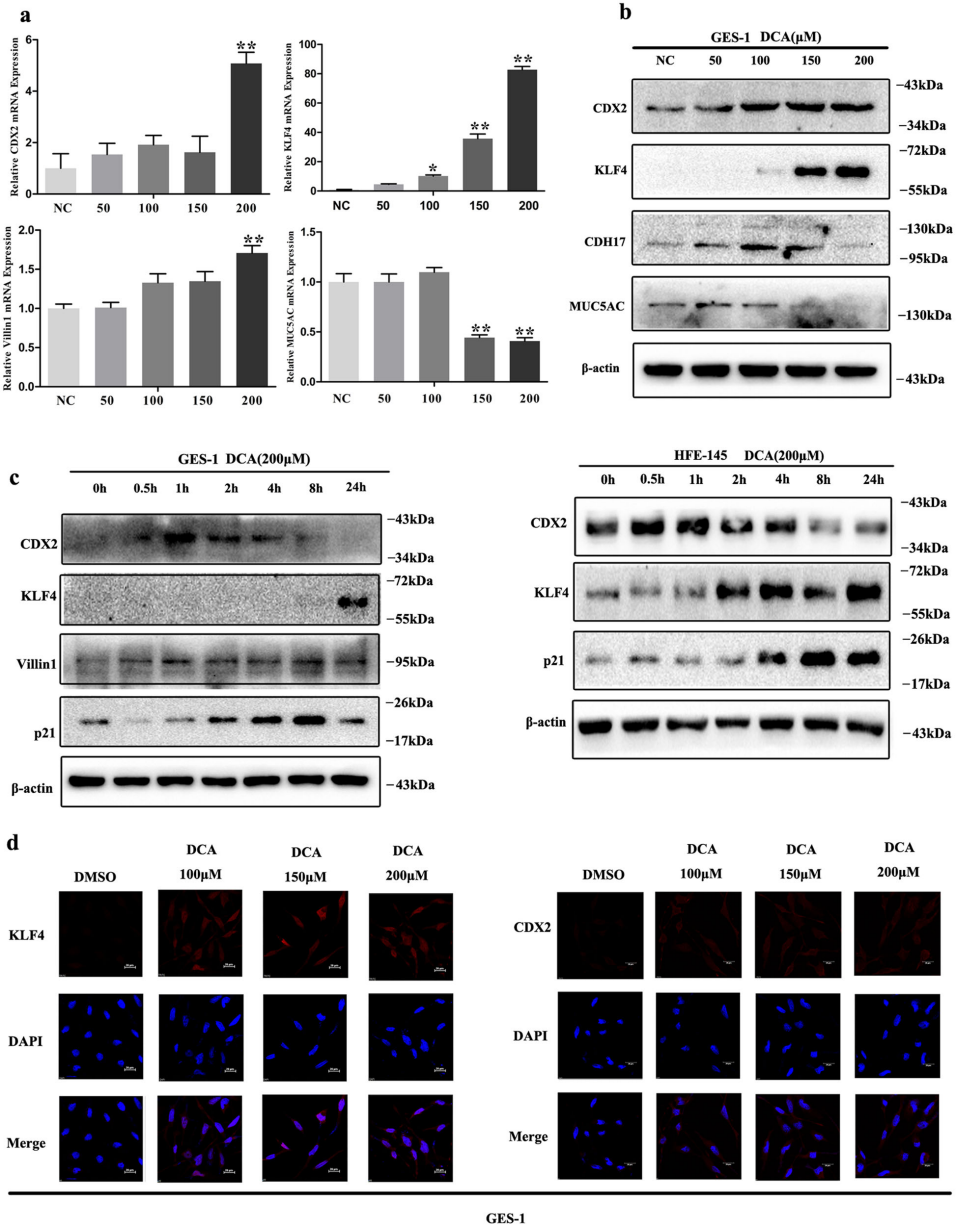
Deoxycholic acid (DCA) treatment induced metaplasia markers expression in gastric epithelial cells. **a**, **b** GES-1 cells were treated with different doses of DCA for 24 h. Next, CDX2, KLF4, Villin1, and MUC5AC mRNA were detected by qRT-PCR. Error bar indicates the SEM, *P < 0.05, **P < 0.01 vs. negative control (NC), *n* = 3. CDX2, KLF4, CDH17, and MUC5AC proteins were detected by western blotting (WB). **c** GES-1 (upper) and HFE-145 (lower) cells were treated with 200 μM DCA in a time-dependent manner. Columnar genes (*CDX2*, *KLF4*, *Villin1*, and *p21*) were examined by WB. **d** GES-1 cells were treated with 200 μM DCA for 24 h. KLF4 (left) and CDX2 (right) expression was analyzed by immunofluorescent staining (red). Nucleus was stained with DAPI (blue). Scale bar, 20 μm.

Herein, we initially detected both columnar and stomach specific genes expression in gastric epithelial cells (Supplementary Fig. S1a, b). Then, both GES-1 and HFE-145, two immortalized human gastric epithelial cell lines, were chosen in the following phenotypic experiments. GES-1, AGS, and BGC823 were chosen in the following gene loss- and gain-of-function experiments. We first treated GES-1 cells with DCA in a dose-dependent manner (0, 50, 100, 150, and 200 μM). The quantitative reverse-transcriptase PCR (qRT-PCR) results showed that DCA treatment could significantly increase KLF4, CDX2, and Villin1 mRNA expression with the highest in 200 μM (Fig. 1a). Furthermore, WB results also showed that KLF4, CDX2, and CDH17 were all increased significantly (Fig. 1b). Simultaneously, DCA treatment significantly inhibited MUC5AC expression in GES-1 cells (Fig. 1a, b). Next, both the GES-1 and HFE-145 cell lines were treated with 200 μM DCA in a time-dependent manner (0, 0.5, 1, 2, 4, 8, and 24 h). The results showed that CDX2, KLF4, Villin1, and p21 were all increased dramatically (Fig. 1c). Finally, GES-1 cells were treated with 200 μM DCA for 24 h and the immunofluorescence (IF) results further revealed increased nucleus expression of CDX2 and KLF4 (Fig. 1d). Together, these results suggest that DCA could induce metaplasia markers and suppress stomach marker in gastric epithelial cells.

### TGR5 promoted metaplasia markers expression and was involved in gastric IM process

**Fig. 2:**
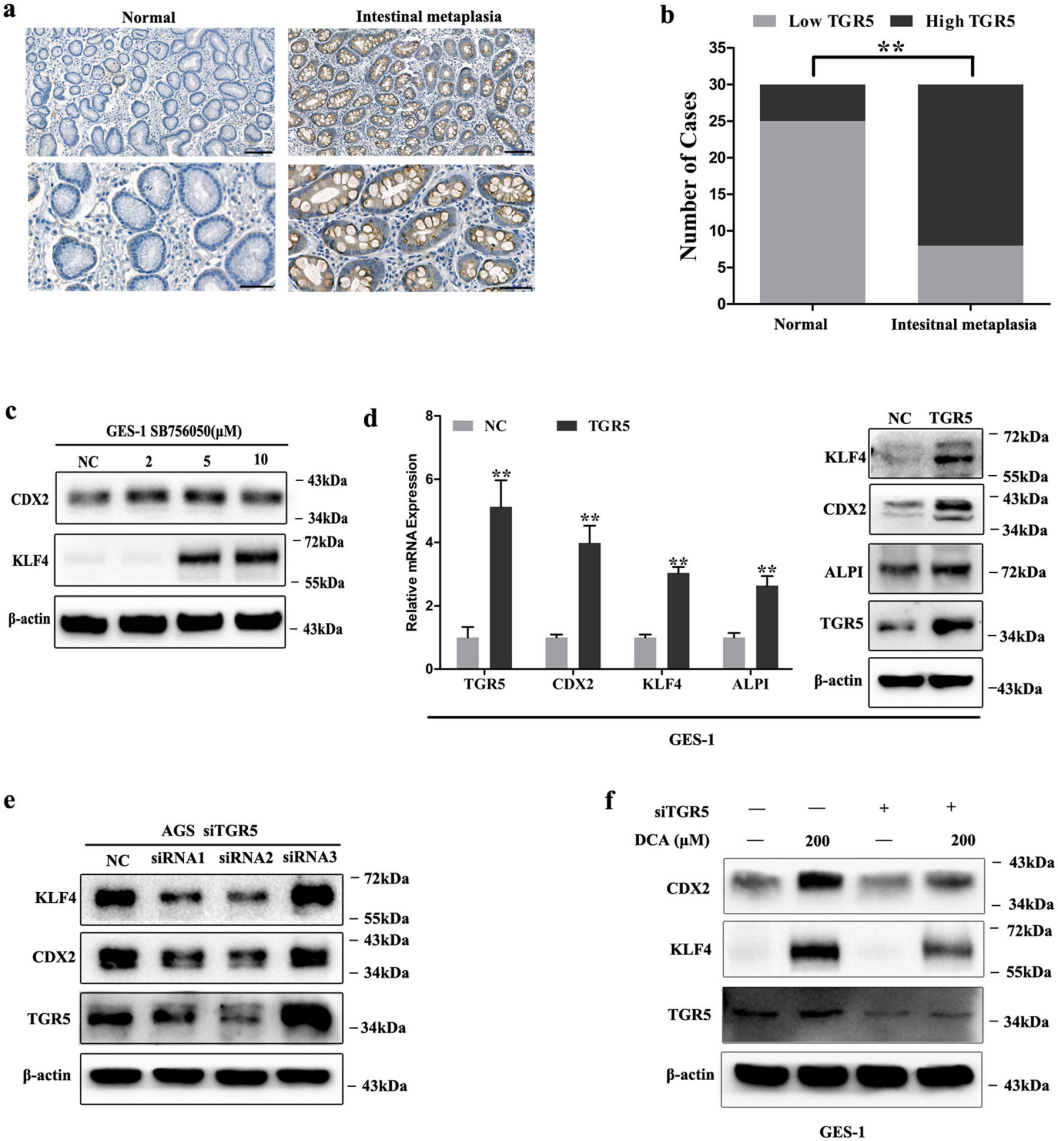
TGR5 was involved in gastric intestinal metaplasia (IM) development. **a** Immunohistochemical staining of normal and IM tissues showing TGR5 expression. Scale bar, 100 μm (upper) and 50 μm (lower). **b** Column charts showed the cases with high and low TGR5 expression in IM tissues. **P < 0.01. **c** GES-1 cells were treated with SB756050 (2, 5, and 10 μM) for 24 h. Then, KLF4 and CDX2 protein expression was examined by WB. **d** GES-1 cells were transfected with TGR5 overexpression lentiviral. TGR5, KLF4, CDX2, and ALPI mRNA and protein levels were analyzed by qRT-PCR and WB. Error bar indicates the SEM, **P < 0.01 vs. NC, *n* = 3. **e** AGS cells were transfected with siRNAs target TGR5 for 72 h. Then TGR5, KLF4, and CDX2 protein expressions were examined by WB. **f** Further, TGR5 was knocked down by transfection with siRNA in GES-1 cells. Next, the cells were treated with DCA (200 μM) for 24 h. TGR5, KLF4, and CDX2 were examined by WB.

TGR5 is the G-protein-coupled receptor-mediated DCA effects^23^. To explore the role of TGR5 in gastric IM process, we initially examined TGR5 expression in normal and IM tissues. IHC results showed significant positive cell cytoplasm and membrane staining of TGR5 in gastric IM tissues compared with normal tissues (Fig. 2a). Further, the cases with high TGR5 expression were significantly more in IM than in normal tissues (Fig. 2b). Then we detected the expression levels of TGR5 in gastric epithelial cell lines (Supplementary Fig. S1c). After that, we treated GES-1 cells with TGR5 agonist SB756050 and results showed that KLF4 and CDX2 expression was significantly increased (Fig. 2c). Further, TGR5 overexpression in GES-1 cells could significantly induce KLF4 and CDX2 expression on both mRNA and protein levels (Fig. 2d). In contrast, we transfected AGS cells with siRNAs target TGR5 and found that KLF4 and CDX2 protein levels were significantly suppressed (Fig. 2e). Lastly, GES-1 cells were transfected with siRNA target TGR5 following DCA treatment (200 μM). The results indicated that TGR5 blocking could significantly alleviate DCA-induced KLF4 and CDX2 expression (Fig. 2f). Together, these results indicate that TGR5 is a key mediator during BA-induced metaplasia markers expression.

### Transcription factors profiling identified HNF4α as a key mediator after DCA exposure

**Fig. 3:**
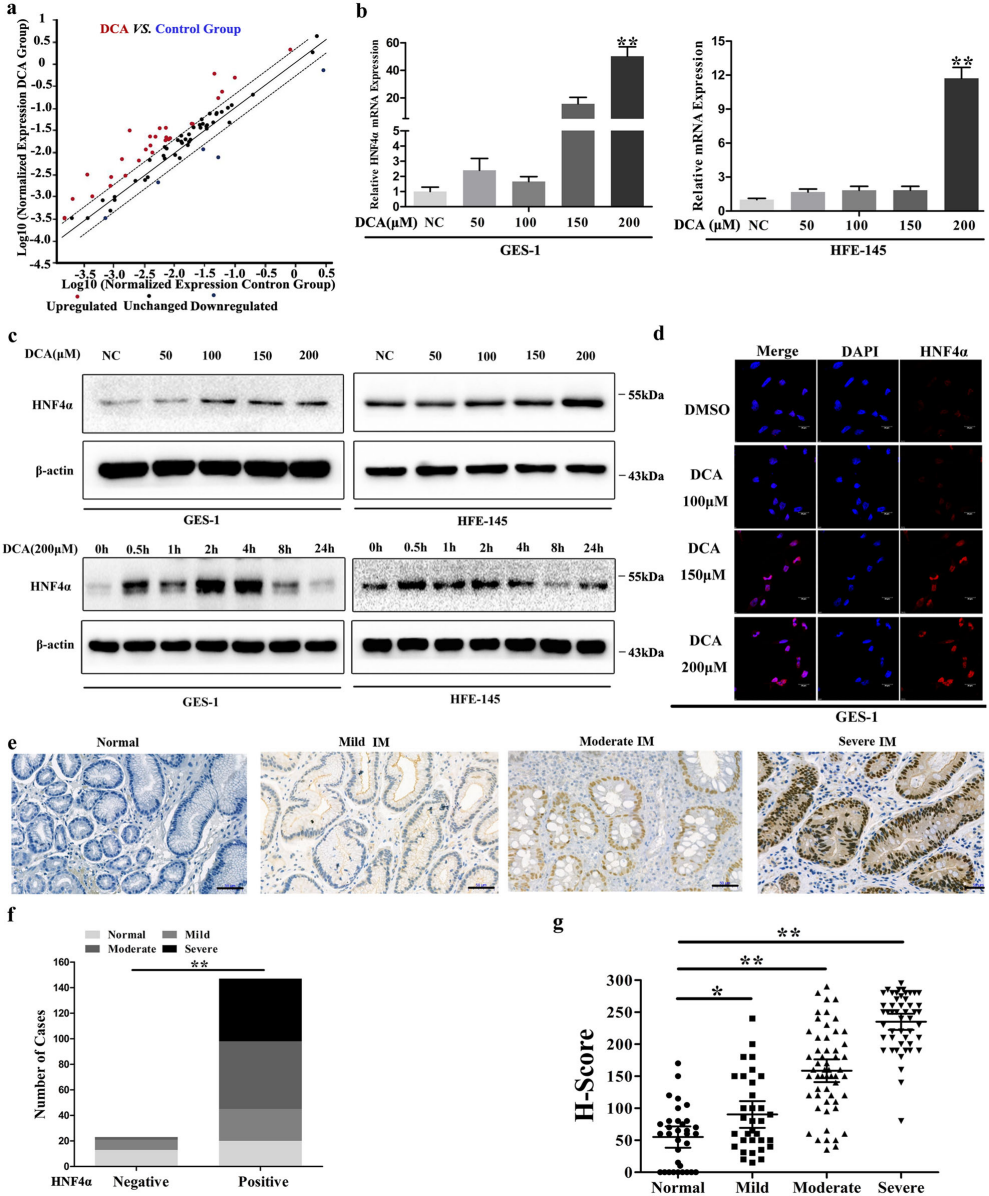
Transcription factors profiling identified HNF4α as a key mediator after DCA exposure. **a** GES-1 cells were treated with 200 μM DCA for 24 h and transcription factor (TF) expression profiles were examined using PCR array. Dots indicated genes over-expressed (red) and under-expressed (blue) (≥2.0 fold) in DCA group vs. Control Group. **b** GES-1 and HFE-145 cells were treated with different doses of DCA for 24 h. HNF4α mRNA levels were examined by qRT-PCR. Error bars indicate the SEM. **P < 0.01 vs. negative control (NC), *n* = 3. **c** GES-1 (left) and HFE-145 (right) cells were treated with DCA in dose- and time-dependent manners. HNF4α protein levels were examined by western blotting. **d** GES-1 cells were treated with 200 μM DCA for 24 h. HNF4α expression was analyzed by immunofluorescent staining (red). Nucleus was stained with DAPI (blue). Scale bar, 20 μm. **e** Immunohistochemical (IHC) staining of normal and IM tissues showed HNF4α expression. Scale bar, 50 μm. **f** Column charts showed the cases with positive and negative HNF4α expression in normal and IM tissues. **P < 0.01. **g** H-scores of HNF4α staining in normal and IM tissues (right). *P < 0.05, **P < 0.01 vs. normal.

To identify the TFs downstream DCA-TGR5 pathway, we treated GES-1 cells with DCA (200 μM) for 24 h and examined differentially expressed TFs using RT2 Profiler PCR Array. Finally, total 46 differentially expressed TFs were detected (fold-change ≥ 2.0 and ≤2.0, 42 upregulated and 4 downregulated) compared with in control cells (Fig. 3a and Table 3).

**Table 3:** Significant changing transcription factors in DCA-treated GES-1 cell line.

Gene symbol	Fold	Description
*ATF3*	6.27	Activating transcription factor 3
*ATF4*	4.73	Activating transcription factor 4
*CEBPA*	4.7	CCAAT enhancer binding protein-α
*CEBPB*	5.07	CCAAT enhancer binding protein-β
*CEBPG*	5.99	CCAAT enhancer binding protein-γ
*E2F1*	2.54	E2F transcription factor 1
*E2F6*	2.69	E2F transcription factor 6
*EGR1*	5.49	Early growth response 1
*ELK1*	2.06	ETS transcription factor ELK1
*ESR1*	2.42	Estrogen receptor 1
*FOS*	3.31	Fos proto-oncogene
*FOXA2*	2.42	Forkhead box A2
*FOXG1*	2.42	Forkhead box G1
*GATA1*	2.42	GATA-binding protein 1
*GATA3*	2.42	GATA-binding protein 3
*GTF2B*	2.19	General transcription factor IIB
*HAND1*	2.42	Heart and neural crest derivatives expressed 1
*HNF1A*	2.42	HNF1 homeobox A
*HNF4A*	2.53	Hepatocyte nuclear factor 4α
*HSF1*	2.53	Heat shock transcription factor 1
*IRF1*	3.71	Interferon regulatory factor 1
*JUNB*	2.19	JunB proto-oncogene
*MAX*	2.34	MYC-associated factor X
*MEF2A*	3.08	Myocyte enhancer factor 2A
*MEF2C*	2.42	Myocyte enhancer factor 2C
*MYC*	3.74	MYC proto-oncogene, bHLH transcription factor
*MYF5*	2.42	Myogenic factor 5
*MYOD1*	2.42	Myogenic differentiation 1
*NFATC2*	2.42	Nuclear factor of activated T cells 2
*NFKB1*	2.4	Nuclear factor-κB subunit 1
*PAX6*	2.42	Paired box 6
*POU2AF1*	2.42	POU class 2 homeobox associating factor 1
*PPARA*	2.66	Peroxisome proliferator activated receptor-α
*PPARG*	3.59	Peroxisome proliferator activated receptor-γ
*RELB*	9.33	RELB proto-oncogene, NF-kB subunit
*SMAD1*	17.72	SMAD family member 1
*STAT1*	12.53	Signal transducer and activator of transcription 1
*STAT2*	4.78	Signal transducer and activator of transcription 2
*TP53*	3.13	Tumor protein p53
*B2M*	2.37	β-2-Microglobulin
*ID1*	−6.67	Inhibitor of DNA binding 1, HLH protein
*SMAD9*	−2.41	SMAD family member 9
*TFAP2A*	−2.48	Transcription factor AP-2α
*ACTB*	−4.34	Actin-β

*DCA* deoxycholic acid.

The Kyoto Encyclopedia of Genes and Genomes (KEGG) pathway analysis by WebGestalt^24^ (http://www.webgestalt.org) showed that, among these changed TFs, maturity onset diabetes in young pathway was most enriched including HNF4α, HNF1α, FOXA2, and PAX6 (Supplementary Fig. S2a). We also used GeneMANIA (https://genemania.org) to visualize the gene networks identified as being enriched (Supplementary Fig. S2b). Accordingly, we focused on HNF4α (≥2.53 vs. control), as previous studies revealed that HNF4α was a pivotal TF in intestine differentiation^25^.

Then we examined HNF4α expression levels in seven gastric epithelial cell lines by WB and qRT-PCR (Supplementary Fig. S1a, b). Next, both GES-1 and HFE-145 cells were treated with different doses of DCA (0, 50, 100, 150, and 200 μM) for 24 h. Both PCR and WB results showed a dose-dependent increase in HNF4α mRNA and protein levels with reaching a maximum at 200 μM (Fig. 3b, c). To further evaluate the DCA-induced kinetic changes in HNF4α expression, GES-1 and HFE-145 cells were stimulated with 200 μM DCA, and the HNF4α protein levels at the indicated time points (0.5, 1, 2, 4, 8, and 24 h) were determined by WB. As shown in Fig. 3c, HNF4α expression increased at as early as 0.5 h following DCA treatment in both GES-1 and HFE-145 cell lines. IF results further revealed HNF4α upregulation in the GES-1 cell nucleus treated with DCA (200 μM) (Fig. 3d). These results clearly demonstrate that DCA exposure efficiently and quickly induces the transcription of HNF4α.

To further determine the effects of BAs on HNF4α activation, a HNF4 TRE clone (Supplementary Fig. S2c) was transfected into GES-1 cells following DCA (200 μM) treatment for 24 h. Remarkably, luciferase activity was induced by nearly twofold compared with negative control (Supplementary Fig. S2d). These results demonstrate that DCA treatment not only increases HNF4α expression but also induces HNF4α transcriptional activity.

We then detected the expression pattern of HNF4α using endoscopic biopsy specimens in normal and IM tissues by IHC (Fig. 3e). The positive rates of HNF4α staining in normal, mild, moderate, and severe IM specimens were 57.6%, 63.6%, 92.7%, and 100%, respectively (*P* < 0.01) (Fig. 3f). The H-score progressively increased from 55.0 ± 8.1 in normal specimens to 235.0 ± 6.3 in severe IM specimens (*P* < 0.01) (Fig. 3g). We next detected the expression patterns of both P1- and P2-HNF4α using gastric tissue microarrays (ST8017a and ST806) including 52 gastritis and 86 IM tissues (Supplementary Fig. S2e). The results indicated that the H-score of P1-HNF4α staining in IM tissues was significantly higher than that in gastritis tissues (106.2 ± 9.3 vs. 20.6 ± 4.9, *P* < 0.01) (Supplementary Fig. S2f). In addition, the H-score of P2-HNF4α in IM tissues was higher than that in gastritis tissues, although no significant difference was noted (*P* > 0.05) (Supplementary Fig. S2f). No significant differences were observed in the H-score between P1-HNF4α and P2-HNF4α in IM tissues (*P* > 0.05) (Supplementary Fig. S2f). These results reveal that both P1- and P2-HNF4α expressions are increased during IM progression, with P1-HNF4α showing a larger increase.

### TGR5-ERK1/2 pathway was required for DCA-induced HNF4α and subsequent metaplasia markers expression

**Fig. 4:**
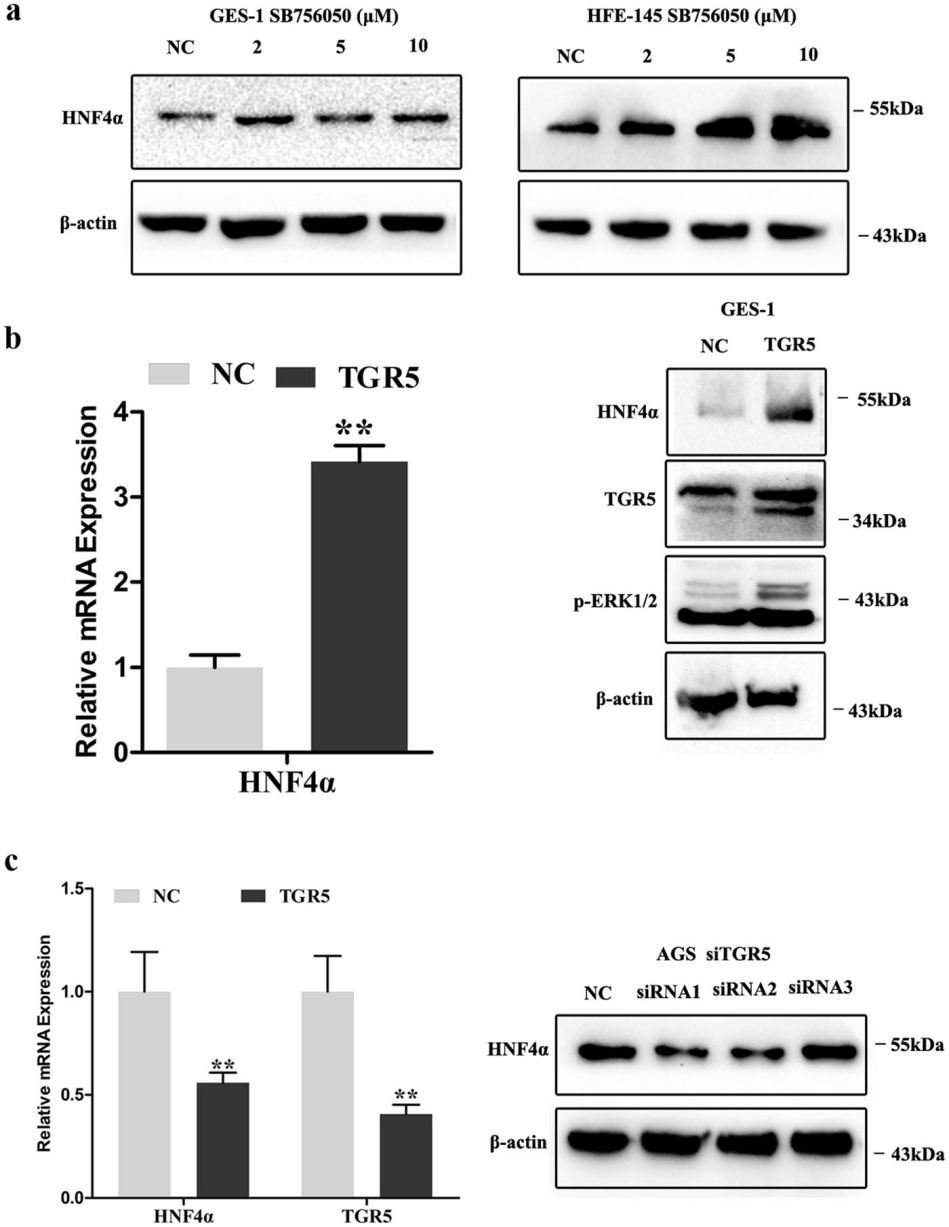
TGR5-mediated bile acids induced HNF4α and columnar genes expression. **a** GES-1 and HFE-145 cells were treated with SB756050 (2, 5, and 10 μM) for 24 h. Then, HNF4α protein expression was detected by western blotting (WB). **b** GES-1 cells were transfected with TGR5 overexpression lentivirus. HNF4α mRNA expression were examined by qRT-PCR. Error bar indicates the SEM, **P < 0.01 vs. negative control (NC), *n* = 3. TGR5, p-ERK1/2, and HNF4α protein expression were examined by WB. **c** AGS cells were transiently transfected with siRNA against TGR5. TGR5 and HNF4α mRNA was examined by qRT-PCR. Error bar indicates the SEM, **P < 0.01 vs. NC, *n* = 3. HNF4α protein expression was examined by WB.

To examine whether TGR5-mediated DCA induced HNF4α, GES-1, and HFE-145, cells were treated with TGR5 agonist (SB756050) for 24 h. We found that HNF4α expression was dramatically induced (Fig. 4a). Then GES-1 cells were transfected with TGR5 overexpression lentivirus and significant increased HNF4α mRNA and protein expression were observed (Fig. 4b). Next, AGS cells were transfected with siRNA against TGR5 for 72 h, revealing that HNF4α mRNA and protein expression were significantly repressed (Fig. 4c).

**Fig. 5:**
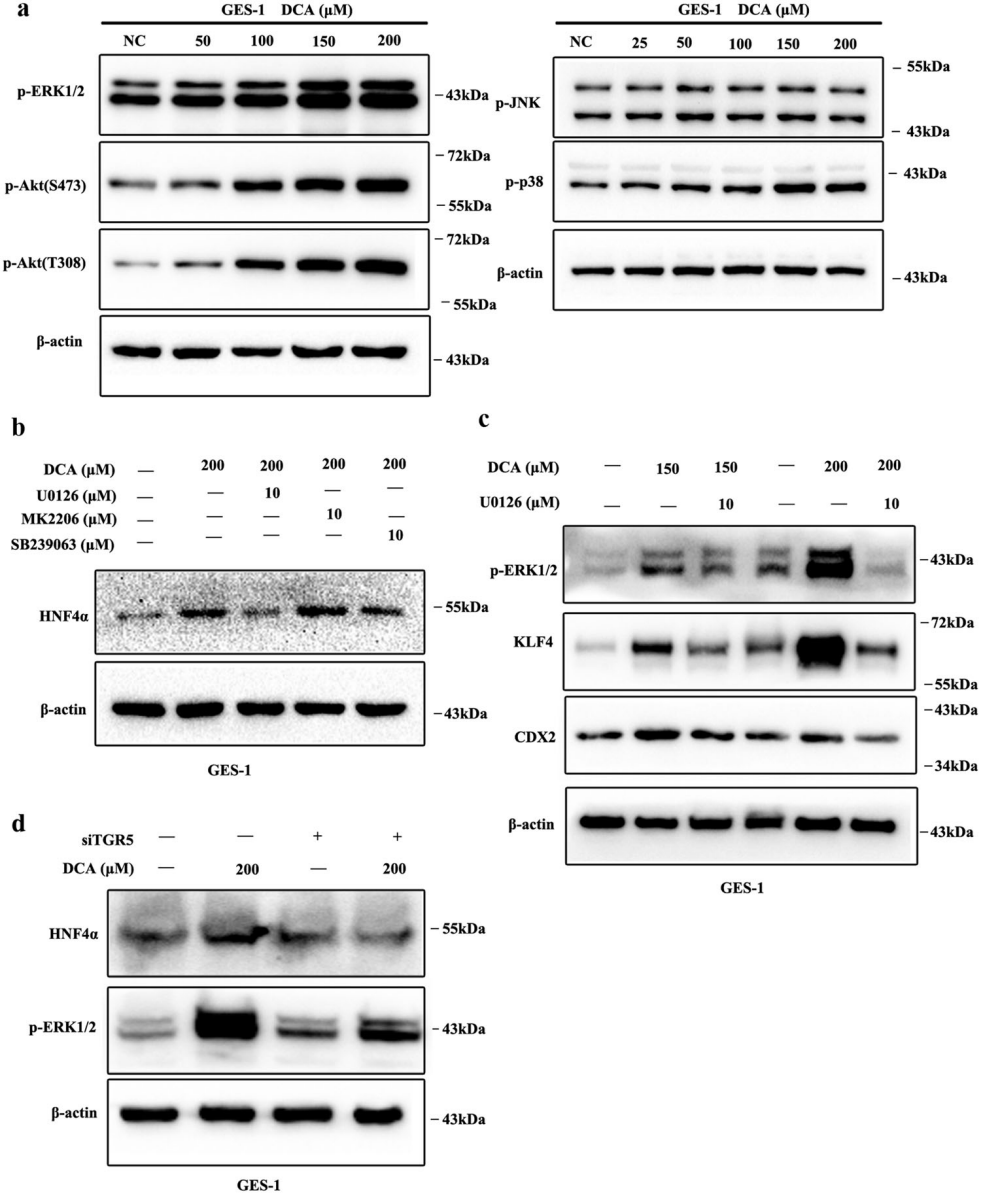
ERK1/2 pathway was involved in DCA-TGR- induced HNF4α and following metaplasia markers expression. **a** GES-1 cells were treated with DCA (0, 25, 50, 100, 150, and 200 μM) for 24 h. Then, MAPK and PI3K/Akt pathways were examined by WB. **b** GES-1 cells were pretreated with U0126 (10 μM), MK2206 (10 μM), and SB239063 (10 μM) for 1 h following DCA (200 μM) treatment for 24 h. Then, HNF4α expression was examined by WB. **c** GES-1 cells were pretreated with U0126 (10 μM) for 1 h following Deoxycholic acid (DCA) (150 and 200 μM) treatment for 24 h. Then p-ERK1/2, KLF4, and CDX2 proteins expression were examined by western blotting. **d** Further, TGR5 was knocked down by transfection with siRNA in GES-1 cells. Next, the cells were treated with DCA (200 μM) for 24 h. p-ERK1/2 and HNF4α were examined by WB.

Previous researches demonstrated that TGR5 could positively regulate mitogen-activated protein kinase (MAPK), PI3K/Akt, and JAK/STAT pathways, and negatively regulate NF-κB, both in physiological and pathological conditions^23^. To determine the pathway-mediated DCA-TGR5 effects, we first treated GES-1 cells with DCA in a dose-dependent manner. Results showed that DCA could significantly induce ERK1/2, p38 MAPK, and PI3K/Akt pathways (Fig. 5a). Then, GES-1 cells were pretreated with ERK1/2 inhibitor U0126 (10 μM), p38 MAPK inhibitor SB239063 (10 μM), and PI3K/Akt inhibitor MK2206 (10 μM) for 1 h before DCA treatment. Results indicated that U0126 could reverse HNF4α expression induced by DCA exposure (Fig. 5b). In addition, pretreatment with U0126 could also significantly block DCA induced p-ERK1/2, KLF4, and CDX2 expression (Fig. 5c). Furthermore, TGR5 overexpression in GES-1 cells could significantly promote p-ERK1/2 expression (Fig. 4b). Lastly, we blocked TGR5 expression using siRNA and then treated GES-1 cells with 200 μM DCA for 24 h. The results indicated that TGR5 silencing significantly alleviated p-ERK1/2 and HNF4α induction by DCA treatment (Fig. 5d). Together, these results indicate that TGR5 and following ERK1/2 pathway is involved in BA-induced HNF4α and metaplasia markers expression.

### HNF4α-mediated DCA-induced columnar genes expression through directly regulating both KLF4 and CDX2

**Fig. 6:**
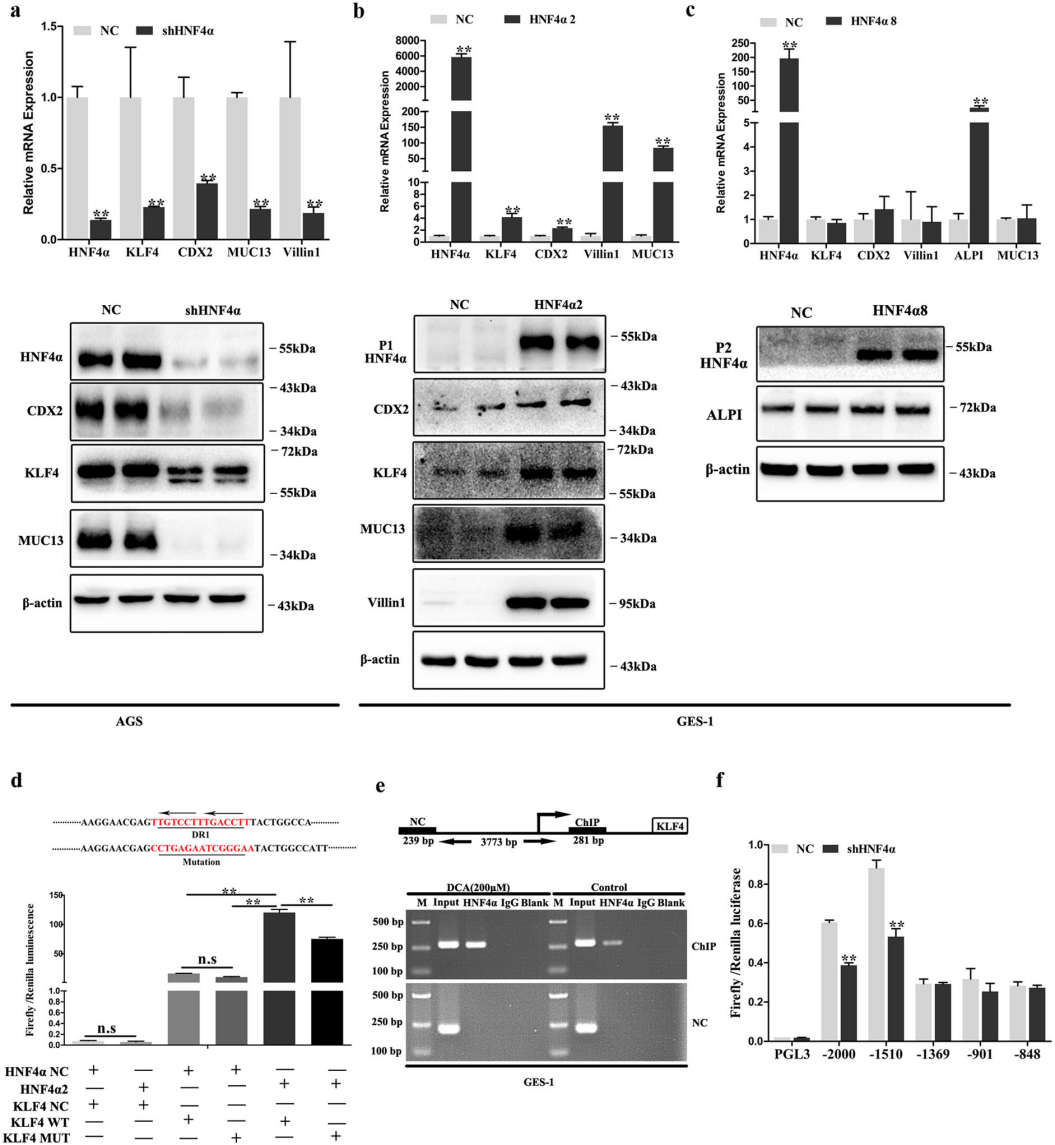
HNF4α transcriptionally regulated KLF4 and CDX2 in gastric epithelial cells. **a** AGS cells were transfected with shRNA lentiviral target HNF4α. Next, P1-HNF4α, P2-HNF4α, CDX2, KLF4, Villin1, and MUC13 were analyzed by western blotting (WB) and qRT-PCR. Error bar indicates the SEM, **P < 0.01 vs. negative control (NC), *n* = 3. **b** GES-1 cells were transfected with HNF4α2 overexpression lentiviral. HNF4α, KLF4, CDX2, MUC13, and Villin1 expression were analyzed by qRT-PCR and WB. Error bar indicates the SEM, **P < 0.01 vs. NC, *n* = 3. **c** GES-1 cells were transfected with HNF4α8 overexpression lentiviral. HNF4α, KLF4, CDX2, MUC13, Villin1, and ALPI mRNA was analyzed by qRT-PCR. Error bar indicates the SEM, **P < 0.01 vs. NC, *n* = 3. ALPI protein level was analyzed by WB. **d** KLF4 promoter fragment (2000 bp) from Ensemble was predicated using JASPAR tool. Reporter constructs containing the predicted HNF4-binding site and mutational site are shown (upper). Negative control (NC) and HNF4α2 overexpression plasmid was transiently transfected with these KLF4 promoter reporter constructs for 24 h and luciferase activity was assayed thereafter (lower). KLF4 promoter activity was expressed as fold induction (means ± SEM) compared with that of NC, *n* = 3. **P < 0.01. **e** GES-1 cells were treated with DCA (200 μM) for 24 h. Then, ChIP assay was performed to demonstrate the direct binding of HNF4α to the KLF4 promoter. DCA, Deoxycholic acid; M, Marker. **f** shHNF4α stably transfected AGS cells were transiently transfected with CDX2 promoter reporter constructs containing the predicted HNF4-binding sites for 24 h and luciferase activity was assayed. CDX2 promoter activity was expressed as the fold induction (means ± SEM) compared with that of NC. **P < 0.01, *n* = 3.

Next, we manipulated HNF4α expression using shRNA in AGS cells and found KLF4, CDX2, MUC13, and Villin1 mRNA and protein expression were all significantly suppressed (Fig. 6a). Furthermore, GES-1 cells were transfected with HNF4α2 (Fig. 6b) and HNF4α8 (Fig. 6c), respectively. Results showed that KLF4, CDX2, Villin1, and MUC13 were upregulated at the mRNA and protein levels after HNF4α2 transfection (Fig. 6d). However, HNF4α8 overexpression only increased ALPI mRNA and protein expression (Fig. 6c). KLF4, CDX2, Villin1, and MUC13 mRNA levels showed no significant differences (Fig. 6c). Then, we directly introduced HNF4α2 in GES-1 cells and the IF results showed significant KLF4 and CDX2 upregulation (Supplementary Fig. S3a, b). These results indicate that both P1- and P2-HNF4α upregulation directly induce columnar genes expression in gastric epithelial cells in a non-redundant manner.

KLF4 have been demonstrated to be a gut-enriched TF, being involved in intestine development, goblet cells differentiation and maturation, and intestinal epithelial homeostasis^26^. Our above results revealed that HNF4α2 could transcriptionally regulate KLF4. Then, we examined the promoter of KLF4 and detected one putative HNF4-binding site (Fig. 6d). Next, we performed luciferase reporter gene analysis and found that HNF4α2 could positively regulate KLF4 promoter activity covering the HNF4-binding site (Fig. 6d). ChIP assays further confirmed that HNF4α bound to the speculative site on the KLF4 promoter in GES-1 cells after DCA treatment (Fig. 6e). Furthermore, we knocked down KLF4 expression in AGS cells and found that CDX2, MUC13, and ALPI mRNA and protein levels significantly decreased (Supplementary Fig. S4a, b), although no significant difference was noted on Villin1 mRNA (Supplementary Fig. S4a).

HNF4α regulates CDX2 expression in both normal intestinal cells and during intestine carcinogenesis^27,28^. We next examined the promoter of CDX2 and detected five putative HNF4-binding sites. To determine the effects of HNF4α on CDX2 promoter activities, we performed luciferase reporter gene analysis and found that HNF4α2 activated the CDX2 promoters between ~1510 and ~2000 bp (Fig. 6f). Then we knocked down CDX2 in AGS cells and introduced CDX2 expression in GES-1 cells. The WB results indicated that CDX2 positively regulated KLF4 and Villin1 expression (Supplementary Fig. S4c, d).

**Fig. 7:**
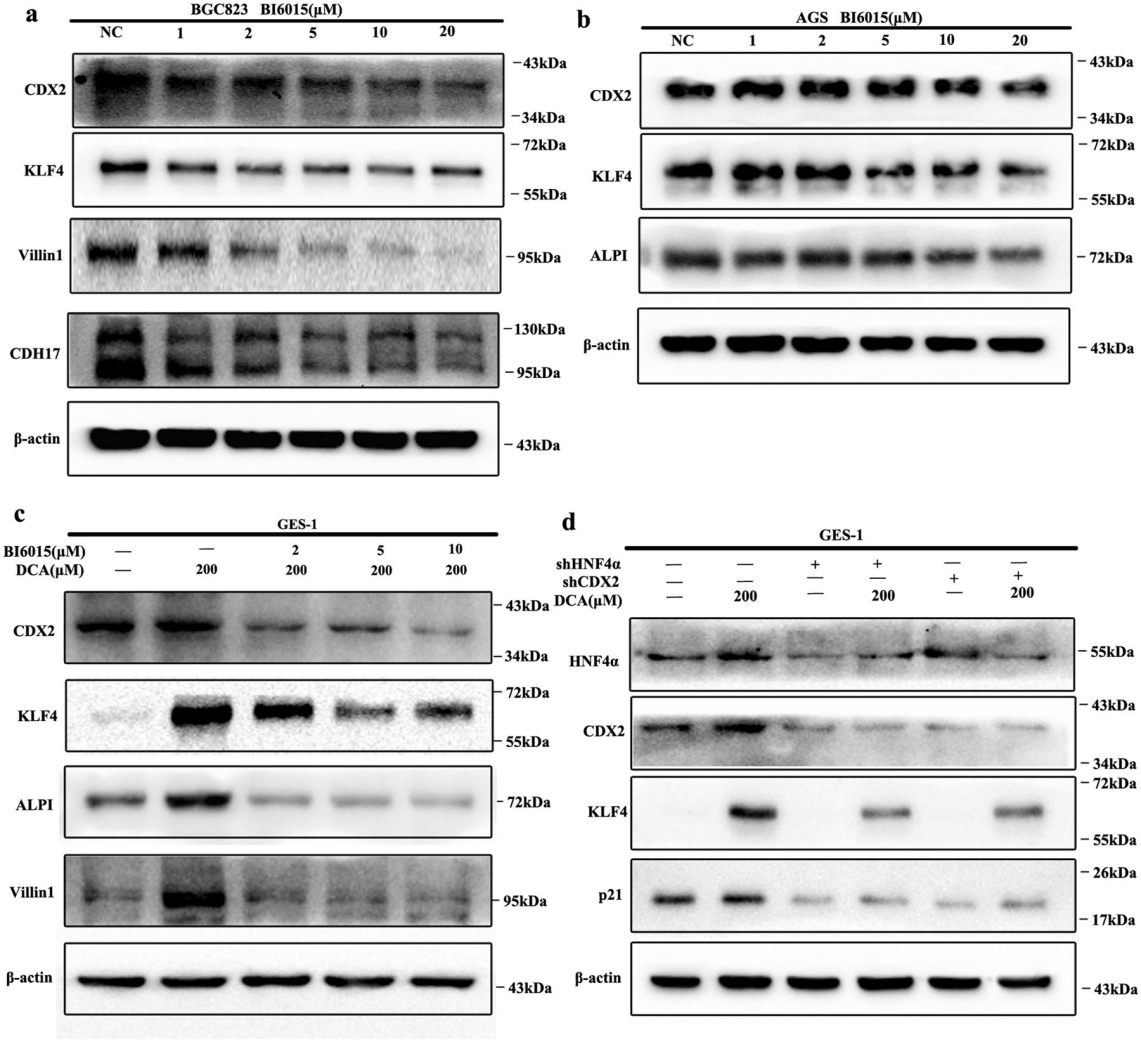
HNF4α-mediated bile acids induced columnar genes expression. **a**, **b** BGC823 cells (**a**) and AGS cells (**b**) were treated with different doses of BI6015 for 24 h. CDX2, KLF4, Villin1, CDH17, and ALPI expression was examined by western blot (WB). **c** GES-1 cells were pretreated with BI6015 (2, 5, and 10 μM) for 1 h and then treated with DCA (200 μM) for another 24 h. KLF4, CDX2, ALPI, and Villin1 expression was detected by WB. **d** HNF4α and CDX2 were knocked down by stable transfection with shRNA lentiviral in GES-1 cells. Next, the cells were treated with DCA (200 μM) for 24 h. HNF4α, CDX2, KLF4, and p21 were examined by WB.

Next, to determine the effects of HNF4α in DCA-induced columnar genes expression, we initially treated AGS and BGC823 cell lines (both with relatively high HNF4α expression) with BI6015, an antagonist of HNF4α^29^, for 24 h and columnar genes expression were examined using WB. The results revealed that BI6015 treatment (1, 2, 5, 10, and 20 μM) significantly inhibited KLF4, CDX2, ALPI, CDH17, and Villin1 expression in a dose-dependent manner (Fig. 7a, b). Next, GES-1 cells were pretreated with BI6015 for 1 h and then treated with 200 μM DCA for 24 h. As expected, pretreatment with BI6015 at 2, 5, and 10 μM reversed DCA-induced KLF4, CDX2, ALPI, and Villin1 expression (Fig. 7c). Lastly, GES-1 cells were successfully transfected with shHNF4α lentiviral and then treated with 200 μM DCA for 24 h. The WB results showed that HNF4α knockdown completely blocked DCA-induced metaplasia markers expression in a similar manner as BI6015 (Fig. 7d).

### Correlation between TGR5 and HNF4α/CDX2/KLF4 in gastric IM tissues

**Fig. 8:**
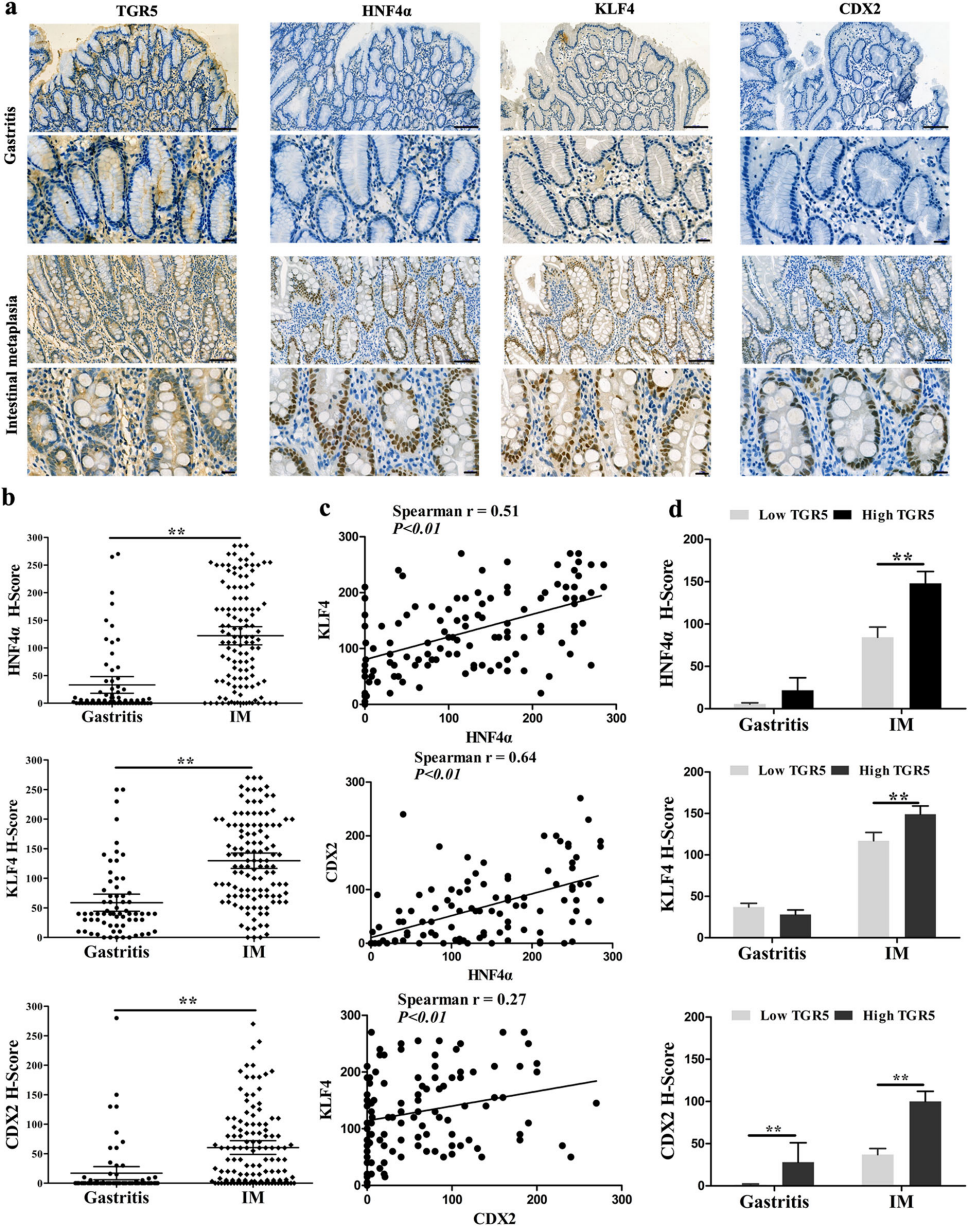
TGR5 expression was positively correlated with HNF4α and metaplasia markers in IM tissues. **a** IHC analysis of gastritis and IM tissues showed TGR5, HNF4α, KLF4, and CDX2-positive staining. Scale bar, 100 μm (upper) and 20 μm (lower). **b** HNF4α, CDX2 and KLF4 H-score in gastritis and IM tissues were compared. Error bars indicate 95% CI. **P < 0.01. **c** Correlation between HNF4α, CDX2, and KLF4 with each other in IM tissues by Spearman’s correlation analysis. **d** HNF4α, CDX2, and KLF4 H-score in gastritis and IM tissues were compared according to high and low TGR5 expression. Error bars indicate the mean ± SEM. **P < 0.01.

To further confirm the relationship between TGR5 and metaplasia markers in IM, we detected TGR5, HNF4α, CDX2, and KLF4 expression using three consecutive slides of gastric tissue including 120 cases IM and 67 cases of chronic superficial gastritis by IHC (Fig. 8a). The H-scores of HNF4α, CDX2, and KLF4 protein were 33.0 ± 7.6 vs. 122.1 ± 8.3 (*P* < 0.01), 17.0 ± 5.6 vs. 60.4 ± 5.9 (*P* < 0.01), and 58.8 ± 7.3 vs. 129.7 ± 6.5 (*P* < 0.01) in gastritis vs. IM tissues, respectively (Fig. 8b). In addition, the expression of HNF4α, CDX2, and KLF4 was positively correlated with each other (*P* < 0.05, Fig. 8c). Moreover, the H-score of HNF4α, KLF4, and CDX2 in high TGR5 cases was significantly higher than in low TGR5 cases in IM tissues (Fig. 8d, *P* < 0.01). These results indicate that HNF4α/KLF4/CDX2 expressions are synchronously increased with TGR5 during IM progression.

## Discussion

Herein, we demonstrated that BAs receptor TGR5 was significantly increased in IM tissues and promoted metaplasia markers expression in gastric epithelial cells. Furthermore, we identified that HNF4α-mediated DCA-TGR5 induced metaplasia markers expression through directly regulating both KLF4 and CDX2 promoter activities. Mechanically, ERK1/2 pathway was involved in DCA-TGR5-induced HNF4α and following metaplasia markers expression.

Molecular changes involved in IM are theoretically recapitulated from the intestine development. Accordingly, key pathways including Wnt, fibroblast growth factor, BMP, Hedgehog, and Notch are involved in Barrett’s esophagus (BE) and IM development^30,31^. In addition, the pivotal TFs involved in intestinal epithelium morphogenesis and differentiation, such as CDX1/2, SOX9, Math1, PDX1, and GATA4/6 are important in IM initiation and evolution through regulating intestine differentiation genes^32^. Intestinal stem cells show stomach features after CDX2 knockdown^33^. KLF4 is a zinc-finger TF primarily in post-mitotic, terminally differentiated epithelial cells in gastrointestinal tract^26^. KLF4^−/−^ mice show a dramatical decrease in goblet cell number and abnormal goblet cell morphology^34^. Although the key roles of CDX2 and KLF4 in metaplasia process have been reported, the underlying mechanism remains unclear.

DGR has been identified as a key risk factor for GC development^35^. In addition, higher concentrations of DCA (400 μM) could promote gastric cells apoptosis^36^ and 200 μM was used to examine IM^37^. The nuclear receptor farnesoid X receptor (FXR) and G-protein-coupled receptor TGR5 mediate the effects of BAs^23,38^. Our previous study showed that primary BAs (CDCA) could promote CDX2 expression through the FXR-SHP-NF-κB^16^ and FXR-miR-92a-FOXD1-NF-κB^10^ signaling pathways. However, the functions of TGR5 in IM have not been fully clarified. TGR5 has been demonstrated to be involved in immune, inflammation, and metabolic disorders^23^. Guo et al.^39,40^ previously showed that TGR5 could suppress gastric inflammation and GC cells proliferation through inhibiting NF-κB and STAT3 pathway. In contrast, Cao et al.^41^ demonstrated that TGR5 expression was increased in intestinal-type GC and mediated BA-induced GC cells proliferation. Here we clearly showed that, during gastric IM development, TGR5 expression was significantly increased and promoted columnar gene expression through ERK1/2 pathway. Thus, we speculated that the functions of TGR5 might be tissue-specific and was diverse due to different external stimulus. And upon BAs exposure, TGR5 contributes to IM development. We first demonstrated that TGR5 could positively regulate both KLF4 and CDX2 expression at transcriptional level upon BA treatment. Our results indicated that TGR5 might directly contribute to malignant transformation of gastric epithelial cells upon BAs exposure.

Next, we identified that HNF4α, a more broad TF that emerges earlier during gut development, was dramatically induced downstream DCA-TGR5 pathway. HNF4α is a highly conserved nuclear receptor expressed in the gut, kidney, liver, and pancreas during early development^42^. The *HNF4α* gene uses two separate promoters, P1 and P2, and is generated into α1-α6 and α7-α9, respectively, through alternative splicing^43^. Normal colon epithelial differentiation and goblet cell maturation are dependent on HNF4α^44^. Moreover, HNF4α is not expressed in the normal esophagus, but emerges in BE and directly induces the columnar phenotype in esophageal epithelial cells^45,46^. Importantly, increased HNF4α expression was observed in gastric IM and intestinal-type adenocarcinomas^47^. In addition, a recent study revealed the feasibility of using HNF4α as a therapeutic target in GC^48^. However, in these models, it remains unclear whether HNF4α mediates gastric epithelial cell *trans*-differentiation in response to BAs exposure. Our results preliminary reveal that both P1- and P2-HNF4α are involved in IM development in non-redundant manner. Interestingly, one recent study revealed that the HNF4α isoforms splicing are involved in BE development^49^, with P1-HNF4α increased significantly without P2-HNF4α upregulation. Accordingly, the functions and distribution of P1- and P2-HNF4α may widely vary during the development of different diseases^50^.

Mechanically, we found that HNF4α could directly regulate KLF4 and CDX2 expression. Further, KLF4 and CDX2 could regulate reciprocally and promote columnar genes expression, which was consistent with previous studies in both BE^51^ and IM^52^. Previous studies demonstrated that HNF4α could directly regulate CDX2, both in intestine development and colorectal cancer^27,28^. However, recent studies using esophagus squamous cells and mouse embryonic fibroblasts revealed no notable CDX2 expression after HNF4α introduction^45,53^. The discrepancy may be due to the different cell lines. In addition, HNF4α, KLF4, and CDX2 could positively regulate their own promoter activities^51,54,55^. Together with our results, this HNF4α central network not only initiates the expression of IM-related genes in a synergetic manner, but also promotes IM persistence after pathogenic factor elimination.

In conclusion, we elucidated that BAs treatment could activate TGR5-ERK1/2 pathway following induction of HNF4α expression, which further promoted metaplasia markers expression through direct regulation of KLF4 and CDX2. These results shed light that suppression TGR5-HNF4α signaling cascade maybe a potential therapeutic target for blocking Correa’s cascade progression and GC development.

## Supplementary information

Figure S1

Figure S2

Figure S3

Figure S4

Supplementary Figure Legends
