# Supplementary figures and images for: TGR5-HNF4α axis contributes to bile acid-induced gastric intestinal metaplasia markers expression
Source: Cell Death Discov. 2020 Jul 6;6:56. doi: 10.1038/s41420-020-0290-3 (PMC7338499; doi:10.1038/s41420-020-0290-3)

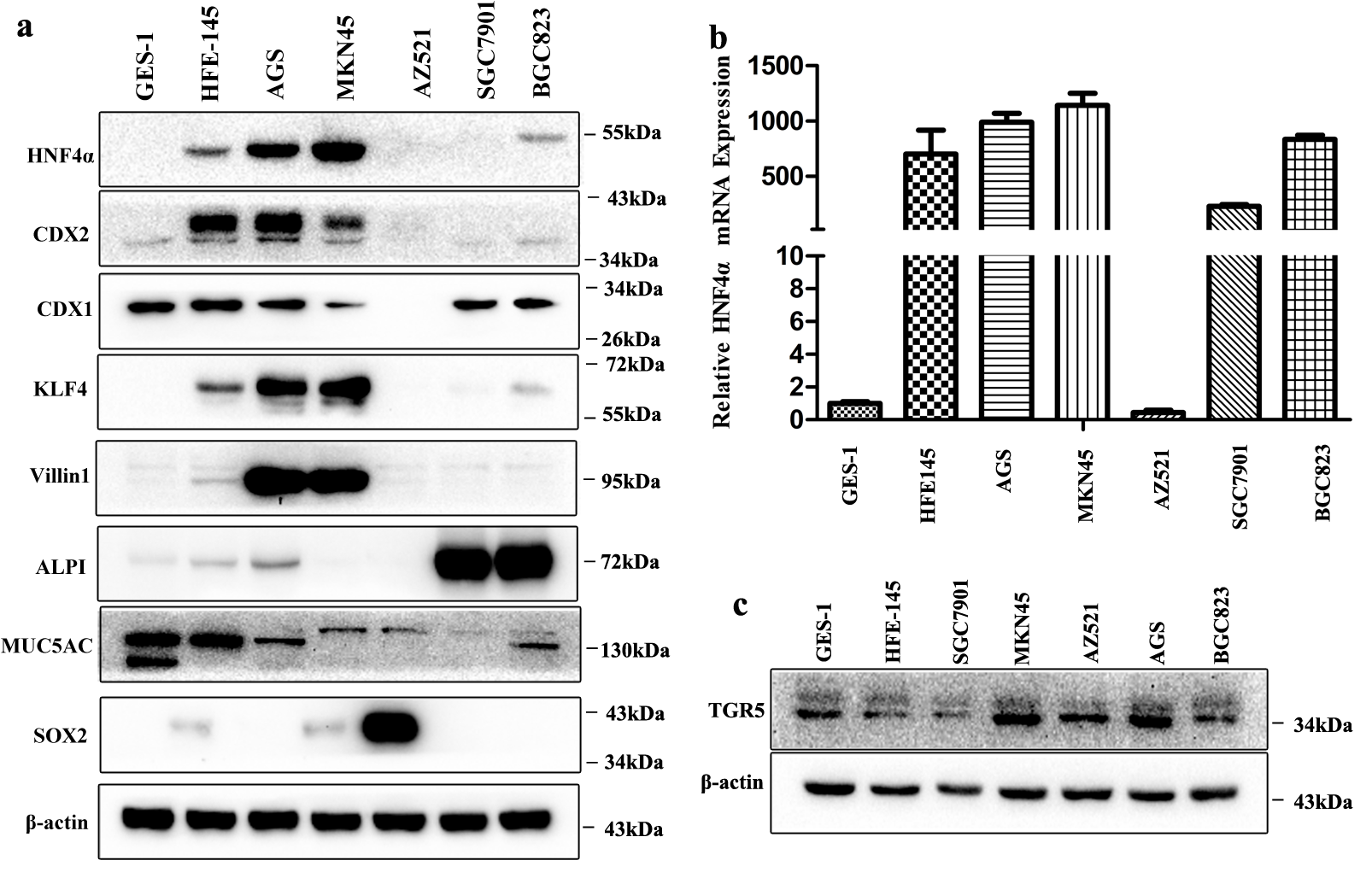

Supplement: Supplementary file 1 — Figure S1 [file 41420_2020_290_MOESM1_ESM.tif]

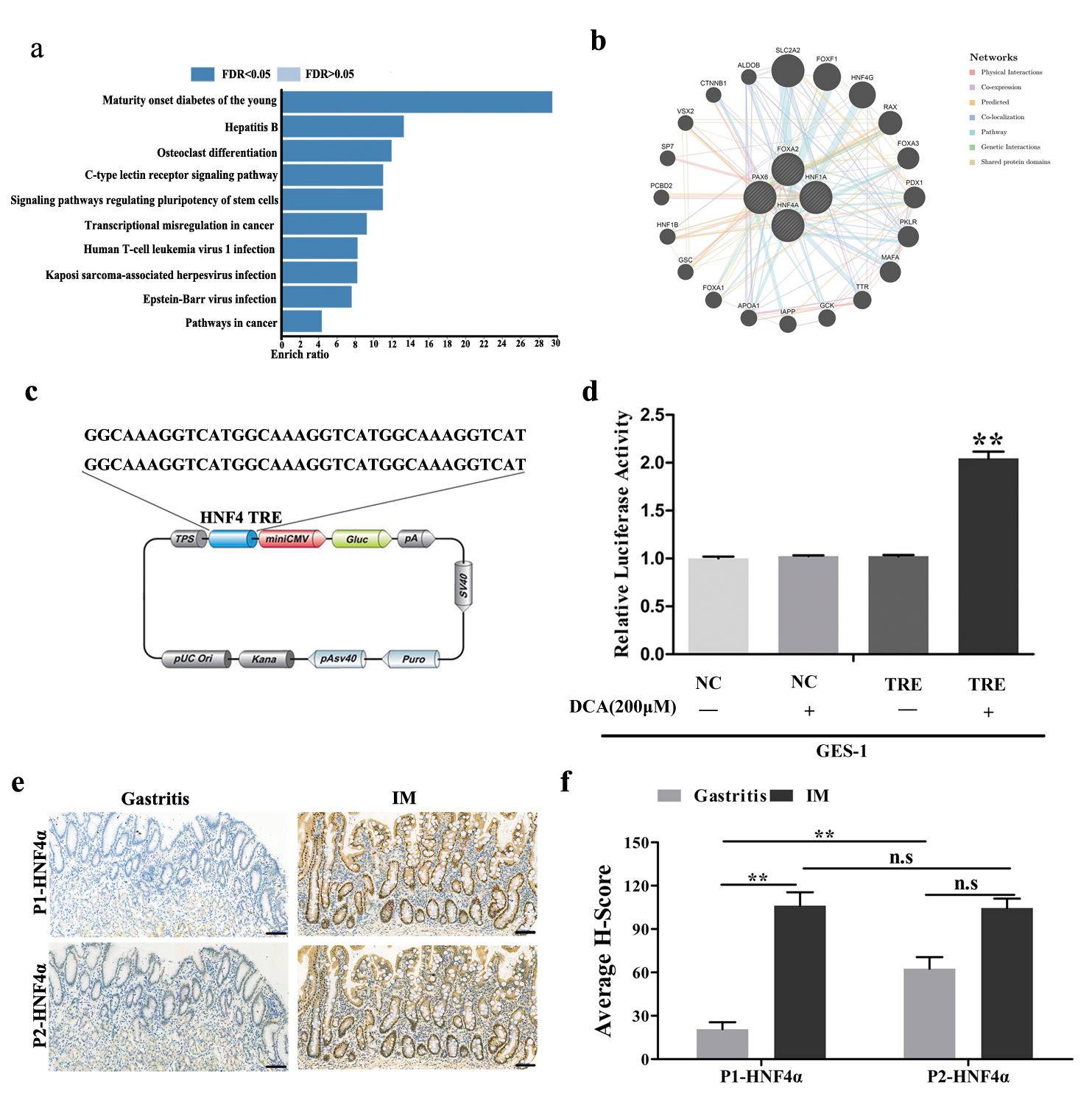

Supplement: Supplementary file 2 — Figure S2 [file 41420_2020_290_MOESM2_ESM.tif]

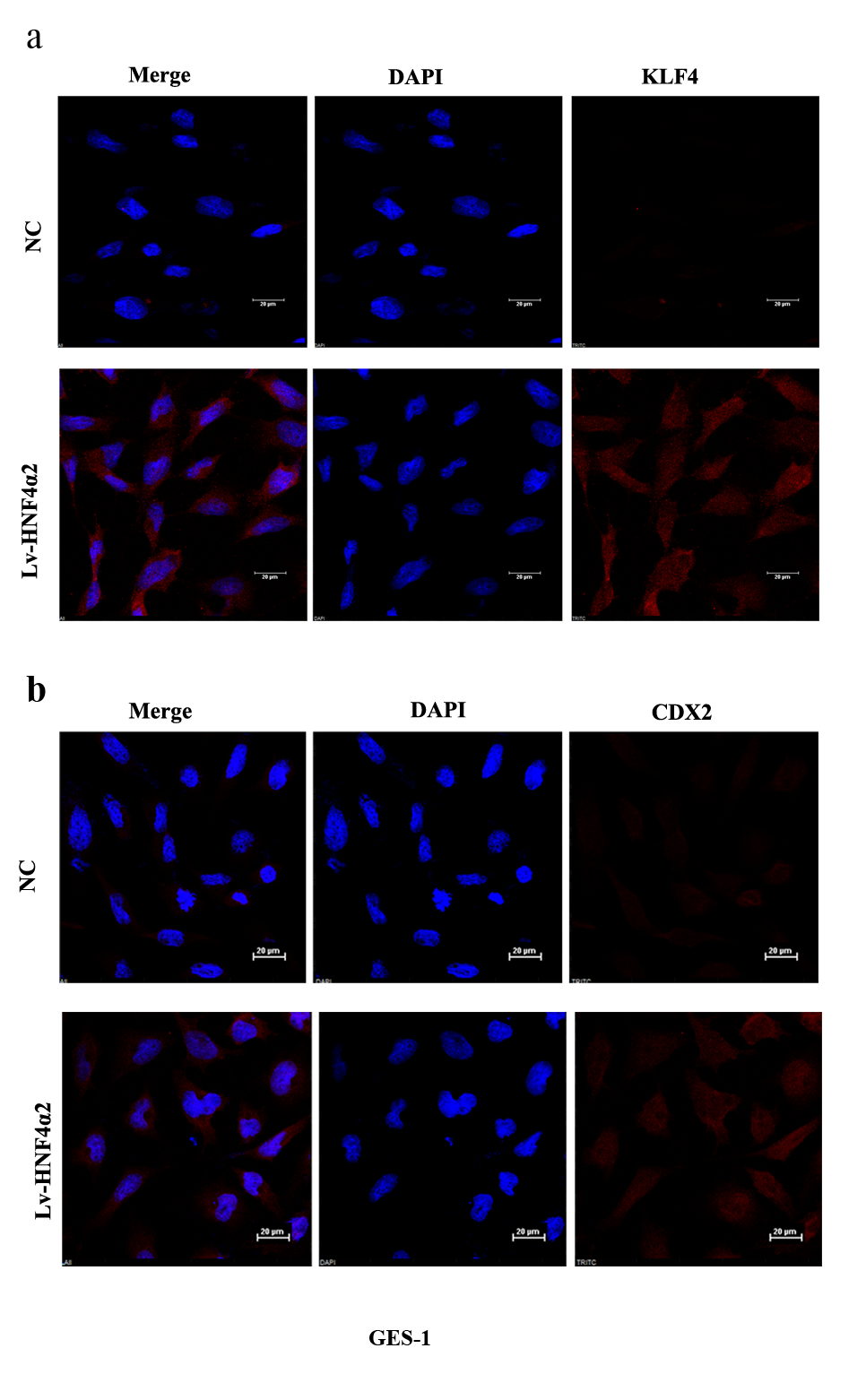

Supplement: Supplementary file 3 — Figure S3 [file 41420_2020_290_MOESM3_ESM.tif]

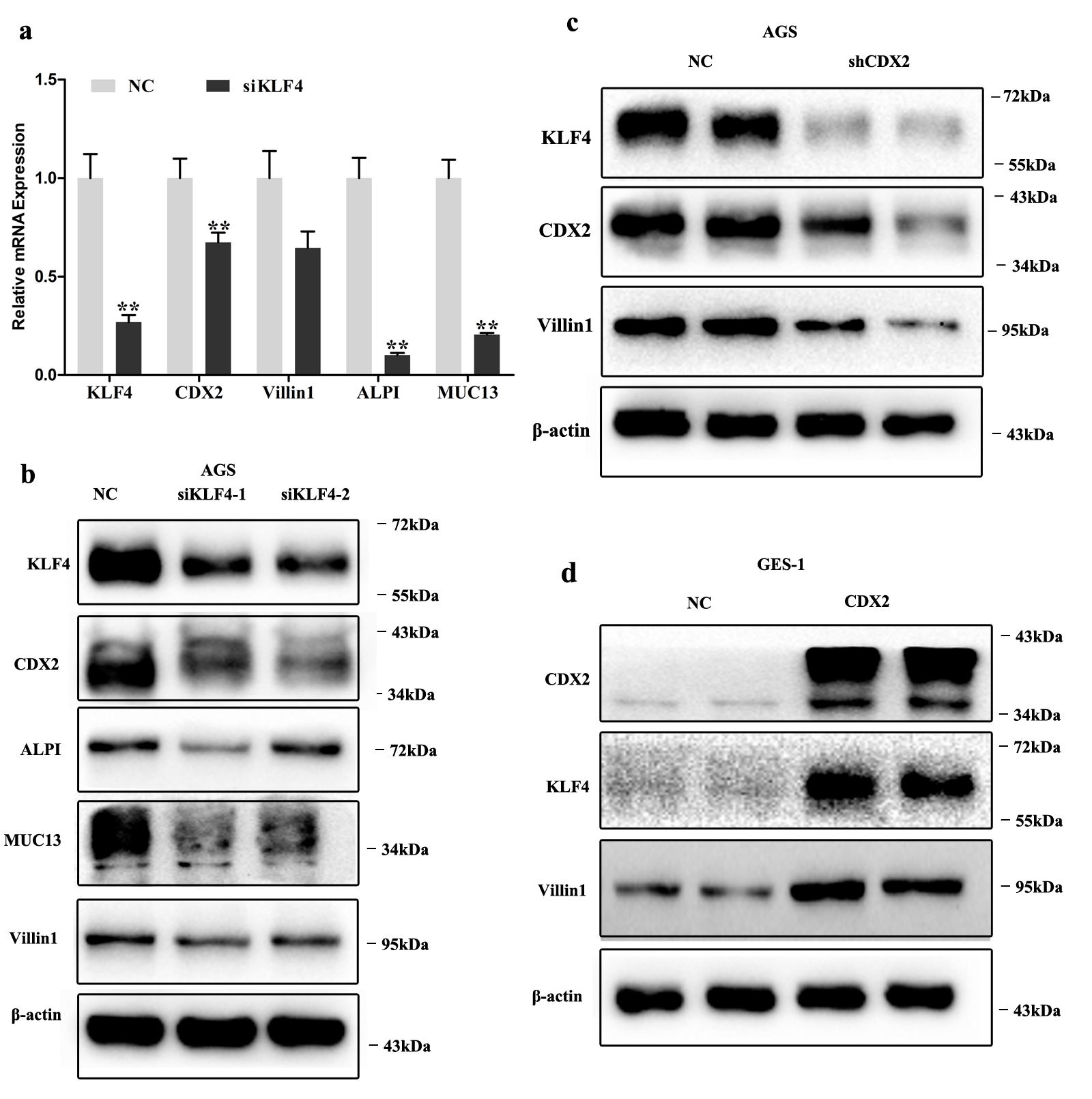

Supplement: Supplementary file 4 — Figure S4 [file 41420_2020_290_MOESM4_ESM.tif]
