# Supplementary material for: TGR5-HNF4α axis contributes to bile acid-induced gastric intestinal metaplasia markers expression
Source: Cell Death Discov. 2020 Jul 6;6:56. doi: 10.1038/s41420-020-0290-3 (PMC7338499; doi:10.1038/s41420-020-0290-3)
Supplement: Supplementary file 5 — Supplementary Figure Legends [file 41420_2020_290_MOESM5_ESM.docx]

**Figure S1.** The expression of HNF4α and TGR5 in gastric epithelial cell lines. **(a)**, The expression of metaplasia and stomach markers in seven gastric epithelial cell lines were analyzed by Western Blot. **(b)**, The expression of hepatocyte nuclear factor-4α (HNF4α) in seven gastric epithelial cell lines were analyzed by qRT-PCR. n=3. **(c)**, The expression of G protein-coupled bile acid receptor 1 (GPBAR1, also known as TGR5) in seven gastric epithelial cell lines were analyzed by Western Blot.

**Figure S2.** The HNF4α expression and transcriptional activity after bile acids treatment. **(a)**. KEEG pathway analysis was conducted by WebGestalt web tool. Bars represent the enrichment ratio of transcription factors in the specified pathway. **(b),** Protein-protein interaction (PPI) network (GeneMANIA) analysis indicating the gene set that was enriched (HNF4α/HNF1α/FOXA2/PAX6). Different colors of the network edge indicate the bioinformatics methods applied: physical interaction, co-expression, predicted, co-localization, pathway, genetic interactions, and shared protein domains. **(c)**, The sequences of HNF4 transcription response element (TRE) used in this study. **(d)**, HNF4 transcriptional response element (TRE) was cloned into the pEZX-PG02 vector. Then GES-1 cells were transfected with 2 μg HNF4 TRE for 24 h and treated with and without 200 μM DCA for another 24 h. Gaussia luciferase activity was detected in the culture supernatant. Empty vector was used as a negative control. ** P < 0.01, n=3. **(e)**. Immunohistochemistry (IHC) staining of gastritis and IM tissues showed P1-HNF4α and P2-HNF4α expression. Scale bar, 100 μm (**upper**) and 50 μm (**lower**). **(f)**, H-scores of P1-HNF4α and P2-HNF4α staining in gastritis and IM tissues. Error bars indicate the mean with 95% confidence interval. ** P < 0.01.

**Figure S3.** P1-HNF4α promoted KLF4 and CDX2 expression in gastric epithelial cells. **(a-b),** KLF4 and CDX2 expression was determined by immunofluorescence (IF) (**Red**) after HNF4α2 introduction in GES-1 cells. Nucleus was stained with DAPI (**Blue**). Scale bar, 20 μm.

**Figure S4.** KLF4 and CDX2 reciprocally regulated to promote columnar genes expression. **(a-b),** siRNAs target KLF4 were transfected into AGS cells. Then CDX2, KLF4, Villin1, MUC13, and ALPI mRNA and protein expressions were examined by qRT-PCR and Western blot (WB). ** P < 0.01 vs. NC, n=3. **(c)**, CDX2 was knocked down in AGS by shRNA. Then CDX2, KLF4, and Villin1 protein expressions were examined by WB. **(d)**, CDX2 overexpression lentivirus were transfected into GES-1 cells. Then CDX2, KLF4, and Villin1 protein expressions were examined by WB.
